# Identification and validation of novel risk genes for intervertebral disc disorder by integrating large-scale multi-omics analyses and experimental studies

**DOI:** 10.3389/fmed.2025.1698050

**Published:** 2025-11-12

**Authors:** Zhe Zhang, Yu Chen, Qianjin Wang, Zheng Li, Bingyang Dai, Cheng Dong, Zhengya Zhu

**Affiliations:** 1 Department of Orthopaedics and Traumatology, Faculty of Medicine, The Chinese University of Hong Kong, Hong Kong SAR, China; 2 Department of Orthopaedics, The Affiliated Hospital of Xuzhou Medical University, Xuzhou, Jiangsu, China; 3 Division of Spine Surgery, Department of Orthopaedic Surgery, Nanjing Drum Tower Hospital, Affiliated Hospital of Medical School, Nanjing University, Nanjing, China; 4 Department of Orthopaedics, The Affiliated Hospital, Southwest Medical University, Luzhou, Sichuan, China; 5 Department of Biomedical Engineering, Faculty of Engineering, The Hong Kong Polytechnic University, Hong Kong, Hong Kong SAR, China; 6 The Hong Kong Polytechnic University Shenzhen Research Institute, Shenzhen, China

**Keywords:** intervertebral disc disorder, transcriptome-wide association study, proteome-wide association study, genome-wide association study, validation study

## Abstract

**Introduction:**

Although genome-wide association studies (GWAS) have identified multiple genetic loci linked to intervertebral disc disorder (IDD), their functional characterization remains largely unelucidated. We aim to leverage an integrative analytical pipeline to identify novel IDD risk genes from genetic associations and experimentally validate their functional roles.

**Methods:**

We integrated transcriptome-wide association studies (TWAS), proteome-wide association studies (PWAS), expression and protein quantitative trait loci (eQTL and pQTL) colocalization analyses to identify potential causal genes for IDD. Enrichment analysis, expression profiling, protein-protein interaction (PPI) network construction, and druggability evaluation were also performed for the prioritized causal candidates. Subsequently, human intervertebral disc (IVD) tissues spanning degeneration grades and an *in vivo* mouse IDD model were employed to functionally characterize candidate risk genes.

**Results:**

Integrative analysis of TWAS and PWAS with colocalization studies identified 104 genes and 10 proteins exhibiting causal associations with IDD. The identified genes/proteins were enriched in extracellular matrix organization, cellular senescence and collagen formation. Crucially, TMEM190, CILP2, and FOXO3 were demonstrated consistent evidence across TWAS, two independent PWAS datasets, and corresponding colocalization analyses, with CILP2 emerging as a potentially druggable target. Differential expression analysis revealed significant upregulated TMEM190 and CILP2, along with downregulated FOXO3 during IVD degeneration. These results were subsequently confirmed at protein levels in clinical specimens. Mouse model experiments further established that down-regulation of CILP2 alleviated IDD progression.

**Discussion:**

Collectively, this work provides an updated compendium of putative IDD risk genes and delineates pathogenic roles for TMEM190, CILP2, and FOXO3, providing a broad hint for further research on novel mechanism and therapeutic targets for IDD.

## Introduction

Intervertebral disc disorder (IDD) represents a primary etiology of low back pain (1). Both genetic predispositions and environmental risk factors involved in its pathogenesis (2–5). Currently, the treatment of IDD primarily relies on symptomatic management with NSAIDs and surgical interventions for more severe cases. However, symptomatic treatments fail to address underlying disease mechanisms, while surgery entails significant costs, potential complications, and surgical morbidity (6). Therefore, identifying causative genes and developing targeted therapeutic strategies is imperative for IDD management.

Recent GWAS have identified multiple loci associated with IDD, predominantly within non-coding genomic regions (7, 8). These regions exhibit complex regulatory mechanisms and linkage disequilibrium, complicating the identification of underlying causal genes. TWAS coupled with eQTL colocalization address this limitation by linking non-coding disease-associated variants to transcriptional changes. In a TWAS study, genetic predictors of gene expression, specifically cis-eQTLs regulating nearby genes, are identified in reference populations, such as the Genotype-Tissue Expression (GTEx) project. These genetic predictors subsequently impute transcriptomic profiles in GWAS cohorts to identify gene expression levels and disease traits (9). eQTL colocalization analyses determine whether shared causal variants gene expression and disease risk share the same causal variants underlie both gene expression and disease risk, strengthening causal inference for candidate genes (10). While IDD-specific TWAS study remains scarce due to the limited large-scale human transcriptomic datasets of disc tissues, GTEx demonstrates substantial eQTLs conservation across tissues (11, 12). Thus, regulatory variants identified in non-disc tissues may still modulate disc biology and IDD susceptibility.

Complementary to TWAS, PWAS utilizes pQTL data to identify protein-level associations with diseases, providing enhanced mechanistic insight. Recently, large-scale human plasma proteome datasets, including those from the ARIC study and Iceland Biobank, have enabled robust pQTL derivation, facilitating practical PWAS implementation (13). Plasma proteins, which serve as key druggable targets and biomarkers for complex traits, can reflect systemic pathological changes associated with IDD. While PWAS has been applied to other diseases (14–16), its application to IDD remains unexplored. Future integration of PWAS with TWAS and eQTL/pQTL colocalization will enable the identification of disease-causing genes with higher precision, while minimizing confounding effects from horizontal pleiotropy. This multimodal approach will elucidate IDD molecular mechanisms and accelerate development of targeted therapeutics.

**FIGURE 1:**
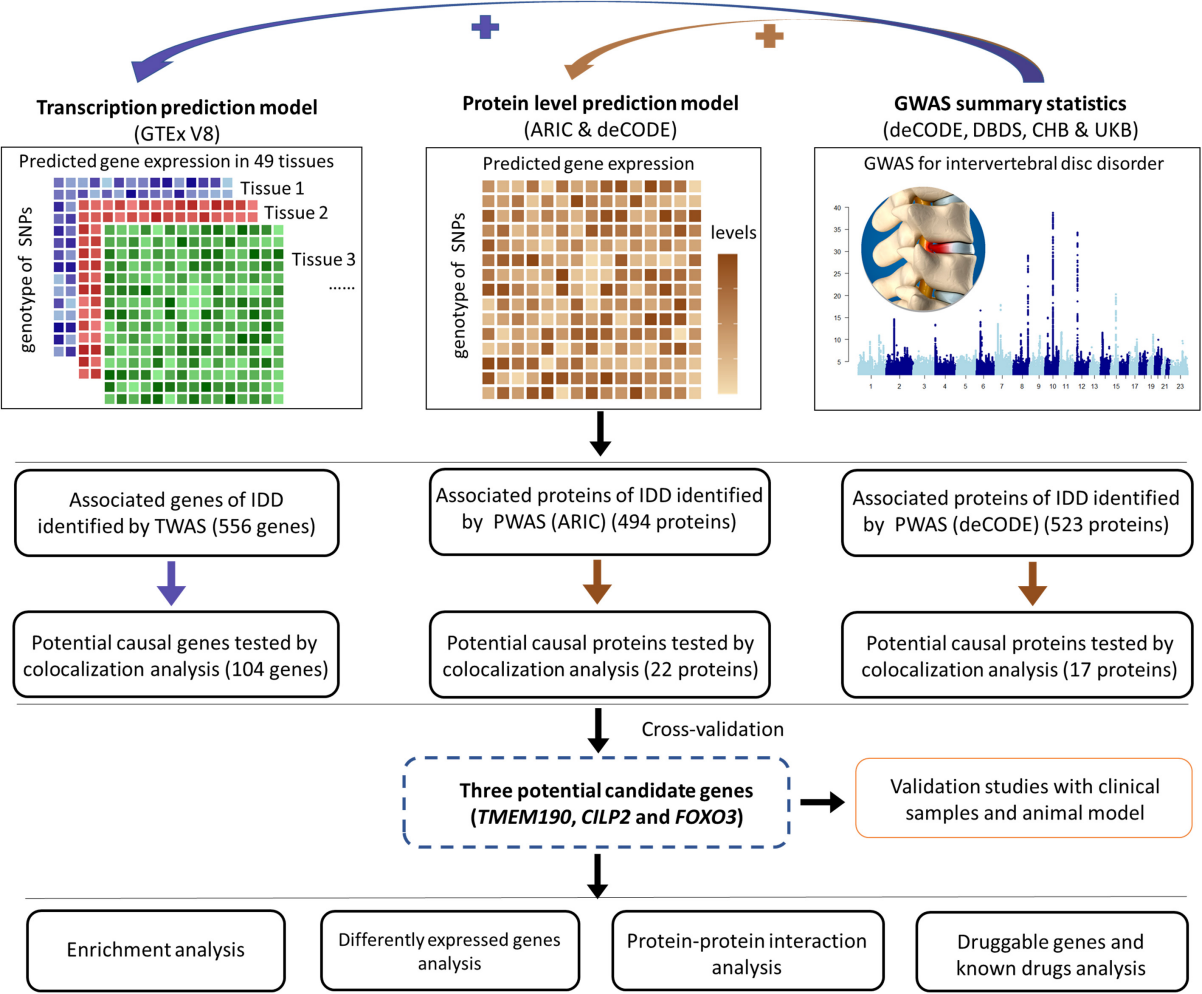
Overview of the study. This schematic illustrates the multistep approach employed to identify potential genes associated with IDD. First, TWAS and eQTL analyses were conducted to identify potential risk genes. Second, two independent PWAS and pQTL analyses were performed to identify potential causal proteins of IDD. Third, enrichment analyses were conducted to elucidate the functions of these potential causal genes/proteins. Fourth, data from the GEO were analyzed to identify differentially expressed potential causal genes. Additionally, PPI analyses were performed to explore interactions among the identified genes. Furthermore, the druggability of the potential causal genes and available drugs that target these genes were explored. Finally, validation studies were performed with clinical samples and an animal model.

This study aimed to identify and validate potential causal genes associated with IDD by integrating multi-omics analyses with experimental approaches. We performed both TWAS and PWAS to uncover novel genes implicated in the pathogenesis of IDD (Figure 1). Colocalization analyses were performed to establish potential causal relationships between these genes and IDD risk. The expression patterns of the prioritized genes in human degenerated intervertebral discs were also assessed. Furthermore, enrichment analysis was conducted to identify key pathways and biological terms associated with IDD. Additionally, we explored protein-protein interactions among the candidates and evaluated their druggability. Finally, experimental validation of top-prioritized genes (TMEM190, CILP2, FOXO3) was conducted using clinical specimens and animal model.

## Materials and methods

### IDD GWAS summary data sources

The GWAS data were obtained from meta-analyses of data from several large cohorts, including deCODE Genetics from Iceland, the Danish Blood Donor Study, the Copenhagen Hospital Biobank, and the UK Biobank (7). Participants provided blood or buccal samples with informed consent, permitting the use of their samples and data in deCODE Genetics and the UK Biobank dataset were included. Each dataset was assumed to share a common odds ratio (OR), while allowing for different population frequencies of alleles and genotypes. Variants with imputation information scores below 0.8 were excluded from the analyses. The GWAS summary data used in our analysis come from the worldcome y data used in These analyses included 58,854 IDD cases and 922,958 controls, with the participants being of European descent. A total of 53.5 million sequence variants were included in the GWAS analysis.

### Transcriptomic data from multiple human tissues

The eQTL data were obtained from GTEx Version 8 (49 tissues) (11). GTEx provides extensive data on the relationship between genetic variation and gene expression, sourced from 838 postmortem donors, and 15,201 RNA-sequencing samples were included, primarily of European descent.

### Human protein abundance references in discovery proteome-wide association studies

The pQTL datasets incorporated in this study were derived from two large-scale investigations: the ARIC study, which includes data on 4,423 proteins from 7,213 individuals (13), and deCODE Genetics, which encompasses 4,428 proteins from 35,559 individuals (17), both primarily of European descent.

### Human intervertebral disc degeneration microarray datasets

Human disc tissue expression microarray datasets were obtained retrospectively from the GEO (GSE56081: *n* = 10, with five degenerative and five normal samples).

### Transcriptome-wide association studies

We performed TWAS analysis by integrating genome-wide summary statistics from an IDD GWAS with eQTL data from GTEx Version 8 across 49 tissue types as descripted before (18). To ensure consistency between datasets, we harmonized the IDD GWAS single nucleotide polymorphisms (SNPs) with the GTEx reference data, aligning SNP reference alleles, effect alleles, and associated metadata. Single-tissue TWAS was conducted for all tissues via SPrediXcan, followed by cross-tissue analysis via S-MultiXcan. S-MultiX can, which is based on a multitissue integration approach, allows for the combination of gene expression data across tissues, enhancing statistical power and enabling the identification of candidate susceptibility genes. We utilized default parameters for the software, with the exception of adjusting the “—cutoff_condition_number” parameter to 30. Only protein-coding genes were considered in the analysis, and significance was determined via a false discovery rate (FDR) threshold of *p* < 0.05.

### FastENLOC colocalization

FastENLOC colocalization tool was used to strengthen the causal inferences drawn from our TWAS findings (19). Briefly, we computed posterior inclusion probability (PIP) values from IDD GWAS data via the torus tool, which quantifies the likelihood of each SNP’s association with IDD. These PIPs were then input into fastENLOC, which performs colocalization analysis by integrating GWAS PIP values with precomputed GTEx multitissue eQTL annotations. Colocalization was performed for each tissue, producing gene-level colocalization probabilities (GLCPs), indicating the likelihood that a given variant influences both IDD GWAS and gene expression in each tissue. The results across all the tissues were then merged, and for each gene, the maximum GLCP value was retained to identify the tissue with the strongest colocalization signal. Genes with a maximum GLCP > 0.5 were considered to have significant evidence of colocalization.

### Proteome-wide association studies

BLISS method was used for PWAS analysis (20). Traditional PWAS approaches rely on individual-level proteomic data, which restricts the use of extensive summary-level pQTL datasets available in public repositories. The BLISS method enables the utilization of large-scale summary-level datasets for more efficient proteomic association analysis by constructing protein imputation models directly from summary-level pQTL data. In this study, we performed PWAS analyses via summary-level pQTL data from two large-scale cohorts: the ARIC study and deCODE Genetics. These datasets include over 4,000 proteins, facilitating a comprehensive analysis of protein–trait associations in the context of IDD. For discovery purposes, proteins with a nominal *p* < 0.05 were considered significant.

### Bayesian colocalization analysis

We also performed Bayesian colocalization analyses via the coloc R package to investigate whether the identified associations between plasma proteins and IDD share the same causal variants rather than being affected by linkage disequilibrium (21). The Bayesian colocalization method evaluated evidence for five distinct hypotheses at each locus: (1) no association with either trait, (2) association with trait 1 only, (3) association with trait 2 only, (4) both traits are associated, but each has distinct causal variants, and (5) both traits share a common causal variant (22). Posterior probabilities for each hypothesis (H0, H1, H2, H3, and H4) were calculated as part of the analysis. Initial prior probabilities were assigned as follows: a SNP exclusively associated with trait 1 (p1) had a probability of 1 × 10−4, a SNP exclusively associated with trait 2 (p2) had a probability of 1 × 10−4, and a SNP shared by both traits (p12) had a probability of 1 × 10−5 (23). A posterior probability of H4 (PPH4) > 0.5 was considered evidence of a shared causal variant between the two traits.

### Enrichment analysis of significant findings

To further investigate the biological role of the identified genes, we performed enrichment analysis via Gene Ontology (GO) categories [encompassing biological processes (BPs), molecular functions (MFs), and cellular components (CCs)], Kyoto Encyclopedia Genes and Genomes (KEGGs) pathways, and Reactome pathways (24, 25). The analysis was performed via the clusterProfiler and Reactome PA R packages. Significant genes or proteins were defined as those with a *p* < 0.05. The background set for the enrichment analysis consisted of all genes or proteins tested in the study, representing the total gene/protein pool from which the significant findings were derived. The ggplot2 R package was used for visualization.

### Annotation of prioritized genes/proteins

The genes/proteins prioritized through TWAS, PWAS, and colocalization were further annotated by evaluating their expression levels in degenerative disc tissues and constructing gene coexpression networks. First, we obtained human disc tissue expression microarray datasets from the GEO (GSE56081: *n* = 10, with five degenerative and five normal samples).^1^ After normalization of the expression matrix, differential expression analysis was performed via the lmFit() and eBayes() functions from the limma package (26). Gene coexpression networks were subsequently constructed to further explore the relationships among the prioritized risk genes, including TMEM190, CILP2, and FOXO3 (15). Briefly, the gene expression matrix of IVD was used to perform correlation analysis between each risk gene and all other genes. The genes were then ranked on the basis of their correlation indices. These correlation coefficients were then used for gene set enrichment analysis (GSEA) with pathway data from Reactome, which was performed via the clusterProfiler package and visualized via the Ridgeplot R package. This approach highlighted significant biological functions and pathways associated with each prioritized gene. Significant enrichment was determined on the basis of an adjusted *p* < 0.05, a normalized enrichment score (|NES|) > 1, and an FDR < 0.25.

### PPI analysis

To investigate potential causal genes implicated in IDD, we employed the STRING database to perform extensive network analysis. The 88 proteins associated with IDD in both TWAS and PWAS were analyzed (Supplementary Data 1). We only reserved connections with an interaction score greater than 0.4.

### Analysis of druggable genes and known drugs

To explore the druggability of potential causal genes of IDD, we conducted druggable gene and known drug analyses. A previous study categorized druggable genes into three tiers (27). We categorized the genes identified via TWAS and eQTL colocalization analyses, as well as genes identified via PWAS (ARIC and deCODE) and pQTL colocalization analyses, and further searched for updated information on the drugs targeting the identified putative causal proteins in the Open Target Platform,^2^ which is a comprehensive tool that promotes drug target identification via the integration of multiple databases.

### Human IVD tissue collection

IVD tissue samples were collected from 6 patients undergoing spinal fusion surgery with the ethics approval of the Affiliated Hospital of Xuzhou Medical University (XYFY2023-KL337-01). The IDD cases were classified according to Pfirrmann’s method (28). Samples falling within the I–II classification were labeled as controls, while falling within the III–V classification were designated as severe IDD samples (29). Patient information is provided in Supplementary Table 1. Informed consent was obtained from all patients.

### Western blot analysis

Total protein was extracted from IVD using a complete cell lysis buffer and quantified with the BCA protein assay kit (Beyotime, China). Protein samples were separated via sodium dodecyl sulfate-polyacrylamide gel electrophoresis (SDS-PAGE) and transferred onto 0.2 μm PVDF membranes (Sigma-Aldrich, United States). The membranes were blocked with a 5% skim milk solution at room temperature and then incubated overnight at 4°C with primary antibodies specific to TMEM190 (1:500; Invitrogen, PA5-70986), CILP2 (1:500; Proteintech, 11813-1-AP), and FOXO3 (1:1,000; Biotime Biotechnology, AF609-1). Following three washes with Tris-buffered saline containing Tween 20 (TBST), the membranes were treated with secondary antibodies at room temperature. Immunoblotting was detected using the UVP ChemiDoc-It Imaging System (UVP, CA, United States) with an enhanced chemiluminescence detection kit (Thermo Fisher; 34,580) applied to the membranes. β-actin served as the loading control, and each blot was analyzed for integrated density using ImageJ software.

### Animal experiments

All experiments were reviewed and approved by the committee of the Institutional Animal Care and Use Committee at Nanjing Drum Tower Hospital (approval number: DWSY-25005637). The methods were described as before (30). Briefly, after anesthesia, 31-G needle was poked into the 8-week-old male C57BL/6 IVD at 90° vertically, rotated 360° and held for 1 min. Sham operation was also performed. These operations were performed on coccygeal IVD. Immediately after the puncture, 2 ul shRNA targeted *Cilp2* gene or negative control encapsulated with the GV112 vector (Shanghai Genechem Co., Ltd.) were injected into the IVD. Four weeks after acupuncture and shRNA injection, the caudal IVD of mice were examined by MRI. Degeneration grade of mice IVD was calculated as described before (28). IVD tissues were then collected for the test of Cilp2 protein levels and histological staining.

### Histological staining and analysis

The harvested IVD tissue was immersed in a 4% paraformaldehyde solution (Solarbio, China) for 48 h to preserve its morphology. It was then decalcified in a 10% EDTA solution (pH 7.2–7.4) for 2 weeks, with daily changes of the solution. The tissue underwent dehydration through a series of graded ethanol baths and was subsequently cleared using an environmentally friendly clearing agent (Solarbio, China). After clearing, the tissue was embedded in paraffin wax and sectioned into 5 μm-thick slices using a microtome. Hematoxylin and Eosin (H & E) staining were applied according to the instructions (Solarbio, China) to observe the histological morphology of the IVD. Finally, histological scoring of the H & E samples was conducted following methodologies described before (31). Simply, the stained results were evaluated from two perspectives, including annulus fibrosus and nucleus pulposus. Each category was assigned a score ranging from 0 to 3, yielding a cumulative score between 0 and 6. Higher scoring levels indicated greater degrees of degeneration.

### Statistical analyses

A chi-square test was conducted to assess the difference in the number of colocalizing genes between IDD-associated and non-associated genes identified by the TWAS. Furthermore, two-tailed *t*-test or one-way ANOVA test were employed to assess: differential expression of TMEM190, CILP2, and FOXO3 in degenerative compared to non-degenerative IVD using GEO dataset, protein levels of these genes in clinical and animal specimens, and degeneration grade, as well as H & E scores in a murine IDD model. Statistical significance (*p* < 0.05) is denoted by asterisks (*) in figures.

## Results

### Identification of genes associated with IDD by TWAS

**FIGURE 2:**
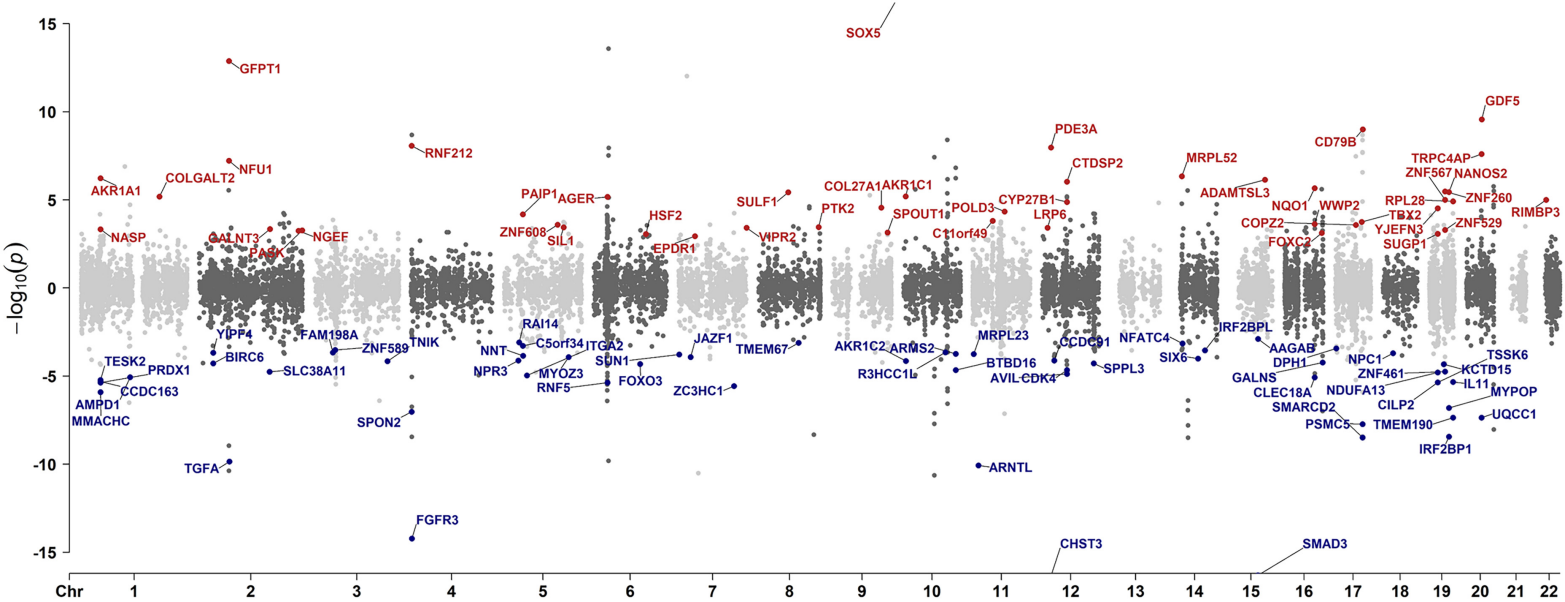
Manhattan plot illustrating TWAS gene associations. Each dot represents a gene plotted according to its genomic position (x-axis) and the significance of the association, measured as the −log_10_(FDR-adjusted *p*-value) (y-axis). Highlighted points with corresponding gene labels indicate genes meeting stringent colocalization criteria: FDR-adjusted *p* < 0.05 and colocalization max-GLCP > 0.5. The color of the highlighted points indicates the directionality of the genetic effect: red represents positive z-mean values (z-mean > 0) and blue for negative z-mean values (z-mean < 0).

As shown in the pipeline (Figure 1), we initiated our analysis by performing a cross-tissue TWAS based on data from reference gene expression predictions from GTEx and the largest intervertebral disc disorder GWAS conducted to date. TWAS analysis of 17,342 protein-coding genes identified 556 significantly IDD-associated genes (FDR-adjusted *p* < 0.05). The top 10 genes most strongly correlated with IDD were *CHST3*, *SOX5*, *SPOCK2*, *SMAD3*, *FGFR3*, *C6orf106*, *GFPT1*, *TWIST1*, *ASCC1*, *IGFBP3* (Figure 2 and Supplementary Data 2). Among the 556 genes, 20 genes, including SMAD3, mapped to nearest genes at the previously reported GWAS susceptibility loci. Our analysis also suggested the novel associations of the remaining 536 genes with IDD risk.

**FIGURE 3:**
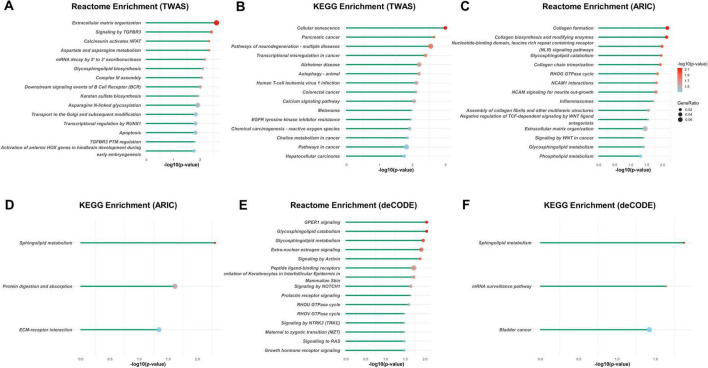
Reactome and KEGG enrichment analyses of the potential associated genes of IDD identified with TWAS and PWAS. Reactome and KEGG enrichment analysis of the potential associated genes of IDD identified with TWAS **(A,B)**, with PWAS using ARIC dataset **(C,D)**, and with PWAS using deCODE dataset **(E,F)**. Each line represents a pathway with significance defined by an FDR-adjusted *p* < 0.05. The color intensity represents statistical significance. The dot size corresponds to the gene ratio, which is defined as the number of genes of a pathway to the total number of genes analyzed.

We then conducted enrichment analysis of the significant findings using the KEGG, Reactome, and GO databases. Notably, the top enriched pathways of TWAS included extracellular matrix organization, cellular senescence, and skeletal system and connective tissue development, all of which established mechanisms in IDD pathogenesis. Other prominent enriched terms included calcineurin activates NFAT, glycosphingolipid biosynthesis, aspartate and asparagine metabolism, cartilage development, response to transforming growth factor-beta, chondrocyte differentiation, regulation of lipid kinase activity, and signal transduction pathways (Figures 3A, B and Supplementary Figure 1).

### Colocalization between IDD risk loci and eQTLs

To determine whether TWAS-identified associations with IDD are driven by shared causal variants, we performed eQTL colocalization analysis using fastENLOC across 49 GTEx tissues for all protein-coding genes. This analysis identified 146 genes with strong colocalization evidence (max-GLCP > 0.5), among which 104 genes were TWAS- prioritized (Figure 2 and Supplementary Data 3, 4). TWAS-prioritized genes showed significant enrichment for colocalization signals compared to a matched background set (χ^2^ = 2195.6, fold-enrichment = 91.7, *p* < 0.001) (Supplementary Table 2), highlighting the specificity and robustness of our findings.

### Identification of plasma proteins associated with IDD by PWAS

**FIGURE 4:**
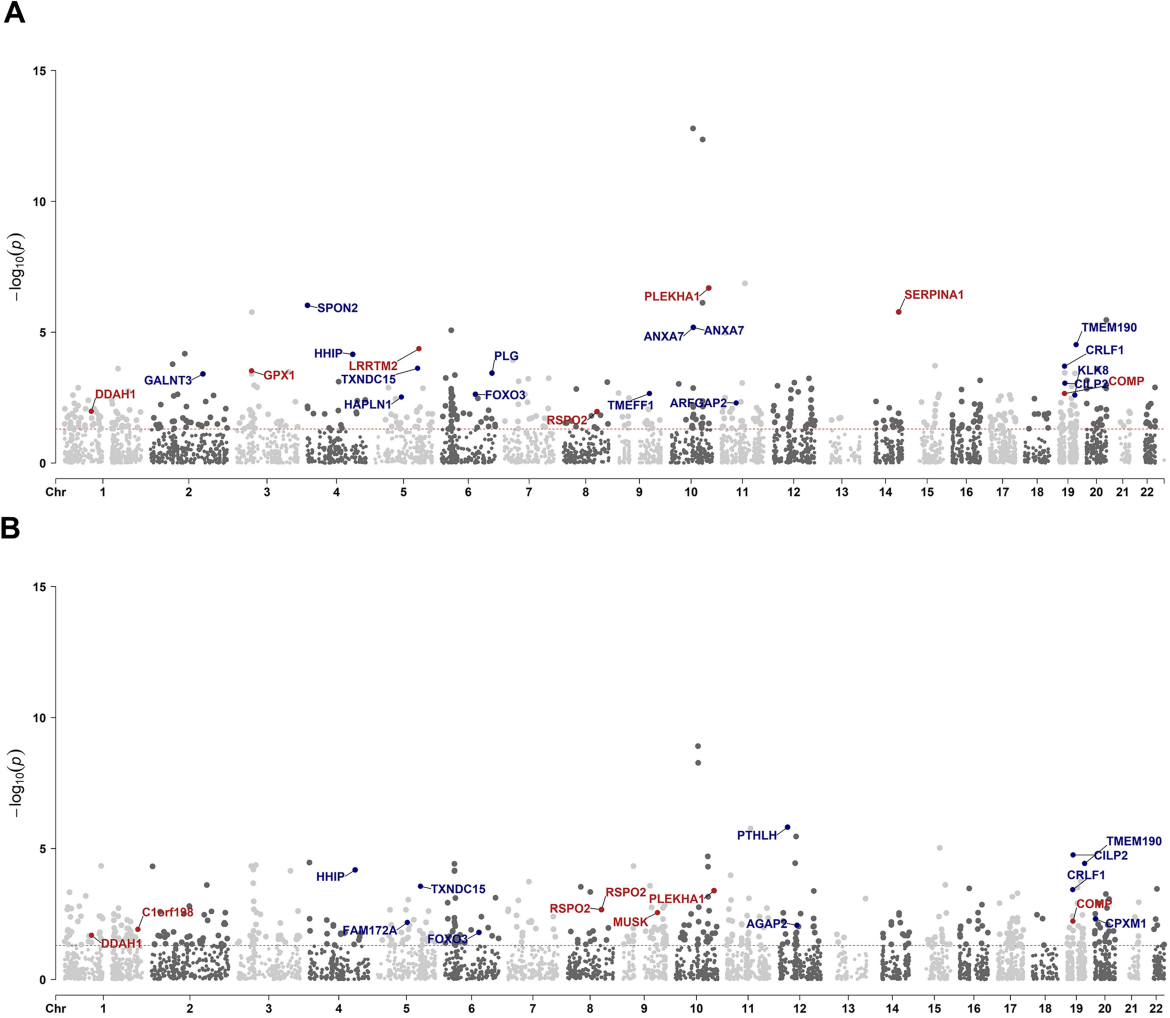
Manhattan plot illustrating PWAS protein associations on the basis of ARIC and deCODE data. Manhattan plot for the ARIC based **(A)** and deCODE based **(B)** PWAS of IDD. Each dot represents a protein plotted according to its genomic position (x-axis), and the significance of the association was measured as the −log_10_(*p*-value) (y-axis). Highlighted points and their protein labels indicate proteins meeting stringent colocalization criteria: *p* < 0.05 and colocalization PPH4 > 0.5. The color of the highlighted points indicates the directionality of the genetic effect: red for positive beta values (beta > 0) and blue for negative beta values (beta < 0).

To identify proteins potentially associated with IDD for further validation, we conducted two independent PWAS by integrating GWAS summary statistics with human plasma proteomic data from the ARIC consortium and deCODE Genetics. The ARIC-based PWAS identified 494 significant associations, and the deCODE-based PWAS yielded 523 associations (Figures 4A, B; Supplementary Data 5, 6) (*p* < 0.05). Among these, 153 proteins were consistently associated with IDD across both datasets (Supplementary Data 7). Among them, six proteins, TMEM190, CILP2, FOXO3, SPON2, GALNT3, and NUF1, were additionally supported by TWAS and eQTL colocalization analyses, further reinforcing their relevance to IDD.

For the PWAS results from the ARIC cohort, the most significant pathways were collagen formation, extracellular matrix organization, and glycosphingolipid/sphingolipid metabolism (Figures 3C, D and Supplementary Figure 2). Similarly, the deCODE PWAS analysis revealed glycosphingolipid/sphingolipid metabolism, regulation of actin cytoskeleton, extra-nuclear estrogen signaling, and signaling pathways associated with GPER1, NOTCH1, PI3K-Akt, and Hedgehog (Figures 3E, F and Supplementary Figure 3). The high degree of consistency between the TWAS and PWAS results across both datasets confirms the robustness of these findings.

### Colocalization between IDD risk loci and pQTLs

To provide causal evidence for IDD-associated proteins, we performed pQTL colocalization analyses. In the ARIC dataset, 26 proteins demonstrated strong colocalization signals with IDD risk loci (PPH4 > 0.5; Supplementary Data 8). Among these, 22 proteins were also significantly associated with IDD in the PWAS (Figure 4A; Supplementary Data 9). In the deCODE dataset, pQTL colocalization identified 24 significant proteins (PPH4 > 0.5; Supplementary Data 10), of which 16 proteins showed consistent PWAS associations with IDD (Figure 4B; Supplementary Data 11). Totally, 10 proteins were identified as causal proteins via ARIC based and deCODE based PWAS and their respective colocalization analyses (Supplementary Data 12). Among these, TMEM190, CILP2, and FOXO3 were also supported by TWAS and eQTL colocalization analysis.

Collectively, TMEM190, CILP2, and FOXO3 emerged as proteins with strong causal evidence for IDD, supported across multiple omics layers including TWAS, two independent PWAS datasets, and both eQTL and pQTL colocalizations (Table 1).

**TABLE 1:** Summary of three potential causal genes of IDD indicated by TWAS, PWAS and colocalization analyses.

		Discovery of TWAS		Validation of PWAS (ARIC)		Validation of PWAS (deCODE)		
Gene	Chr	Max-GLCP	FDR *p*	PPH4	*P*-value	PPH4	*P*-value	Expression
*TMEM190*	19	0.93	1.83 × 10^–5^	0.99	3.00 × 10^–5^	0.99	3.74 × 10^–5^	Up-regulated
*CILP2*	19	0.69	8.62 × 10^–4^	0.92	9.00 × 10^–4^	0.70	1.75 × 10^–5^	Up-regulated
*FOXO3*	6	0.87	0.006	0.79	0.002	0.69	0.016	Down-regulated

### Evaluation of the expression levels of genes/proteins identified by TWAS/PWAS

**FIGURE 5:**
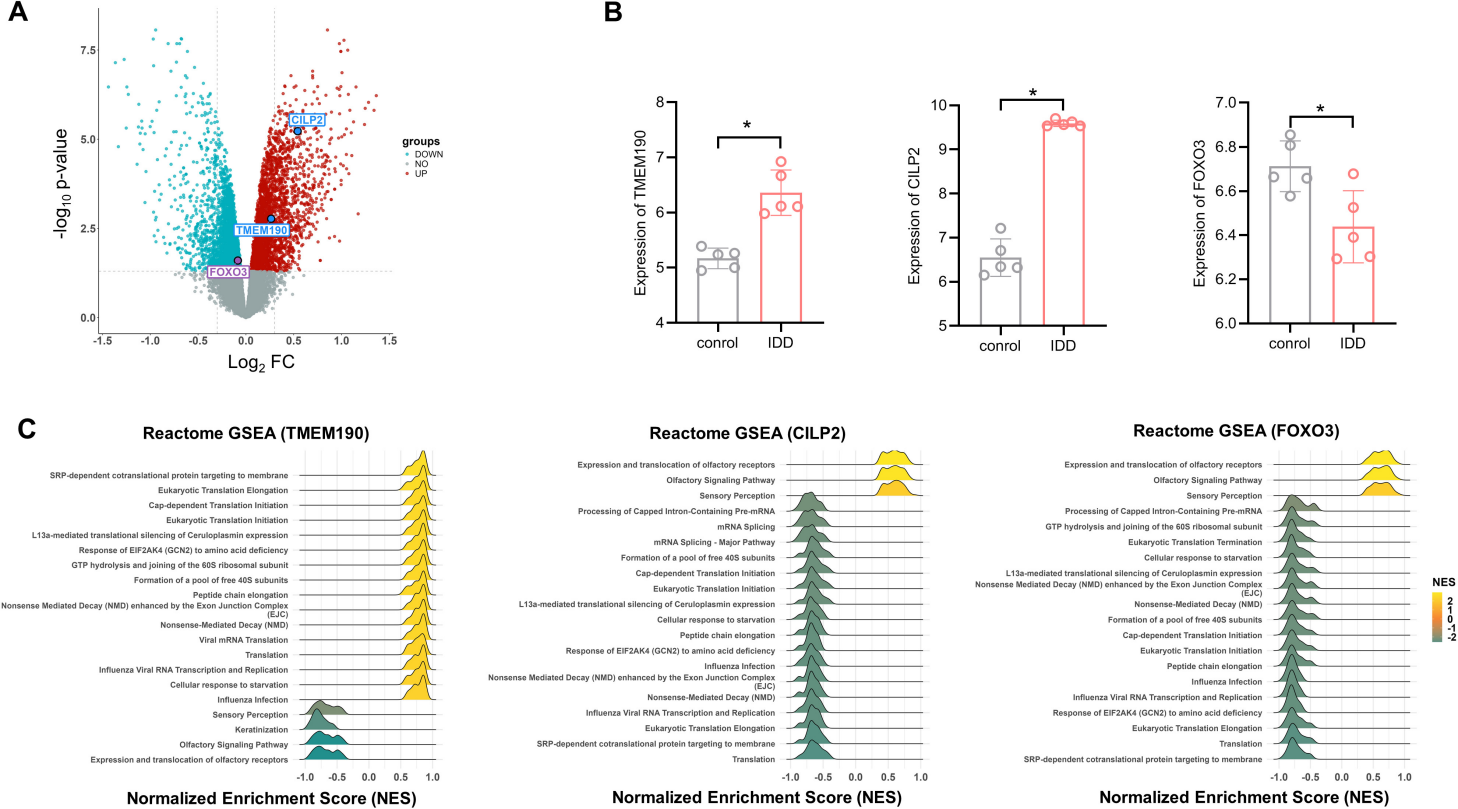
DEGs and enrichment analysis of the three potential causal genes of IDD. **(A)** Volcano plot of the DEG analysis. Each dot represents a gene plotted according to the significance of the association measured as the −log_10_ (FDR-adjusted *p*-value) (y-axis). The colors of the points are as follows: red for upregulated genes, blue for downregulated genes, and gray for non-differentially expressed genes (FDR-adjusted *p* ≥ 0.05). **(B)** Expression levels of potential causal genes for IDD in degenerative vs. non-degenerative groups, based on microassy data from clinical samples *n* = 5. Two-tailed *t*-test. *p < 0.05. **(C)** Significantly enriched pathways for the three potential causal genes (*TMEM190*, *CILP2* and *FOXO3*) as determined by GSEA. Each line represents a pathway with significance defined by an FDR-adjusted *p* < 0.05. Yellow indicates upregulation (NES > 0), while green indicates downregulation (NES < 0).

To identify differentially expressed genes (DEGs) in degenerative intervertebral discs, we analyzed mRNA expression profiles from human disc tissues using the microarray dataset GSE56081. Analysis of the GSE56081 dataset encompassing 13,170 genes captured 537 (96.6%), 395 (80.0%), and 404 (77.2%) of IDD-associated genes/proteins identified by TWAS, ARIC based, and deCODE based PWAS, respectively. Among these genes, 2,877 were significantly upregulated and 3,140 were downregulated in degenerated discs (Figure 5A; Supplementary Data 13). We observed 189 genes overlapped with TWAS-prioritized candidates (Supplementary Data 14) and 53 overlapped with proteins identified in both PWAS analyses (Supplementary Data 15).

In particular, all three genes (*TMEM190*, *CILP2*, and *FOXO3*) priorized by multiple-omics analyses were found to be significantly differentially expressed in degenerative intervertebral discs (*p* < 0.05) (Figures 5A, B and Table 1). Specifically, *TMEM190* and *CILP2* were upregulated in IDD samples, whereas *FOXO3* was downregulated compared to the control group. Notably, *CILP2* showed the most pronounced change, exhibiting a 1.5-fold increase in expression in degenerated discs relative to controls.

### Functional annotation of *TMEM190*, *CILP2*, and *FOXO3*

The three potential causal genes were analyzed within the framework of GSEA to investigate their functions by coexpression analysis (Figure 5C). The GSEA results revealed that all of the three genes involved in the expression and translation of olfactory receptors, sensory perception and SRP-dependent cotranslational protein targeting to the membrane pathways.

**FIGURE 6:**
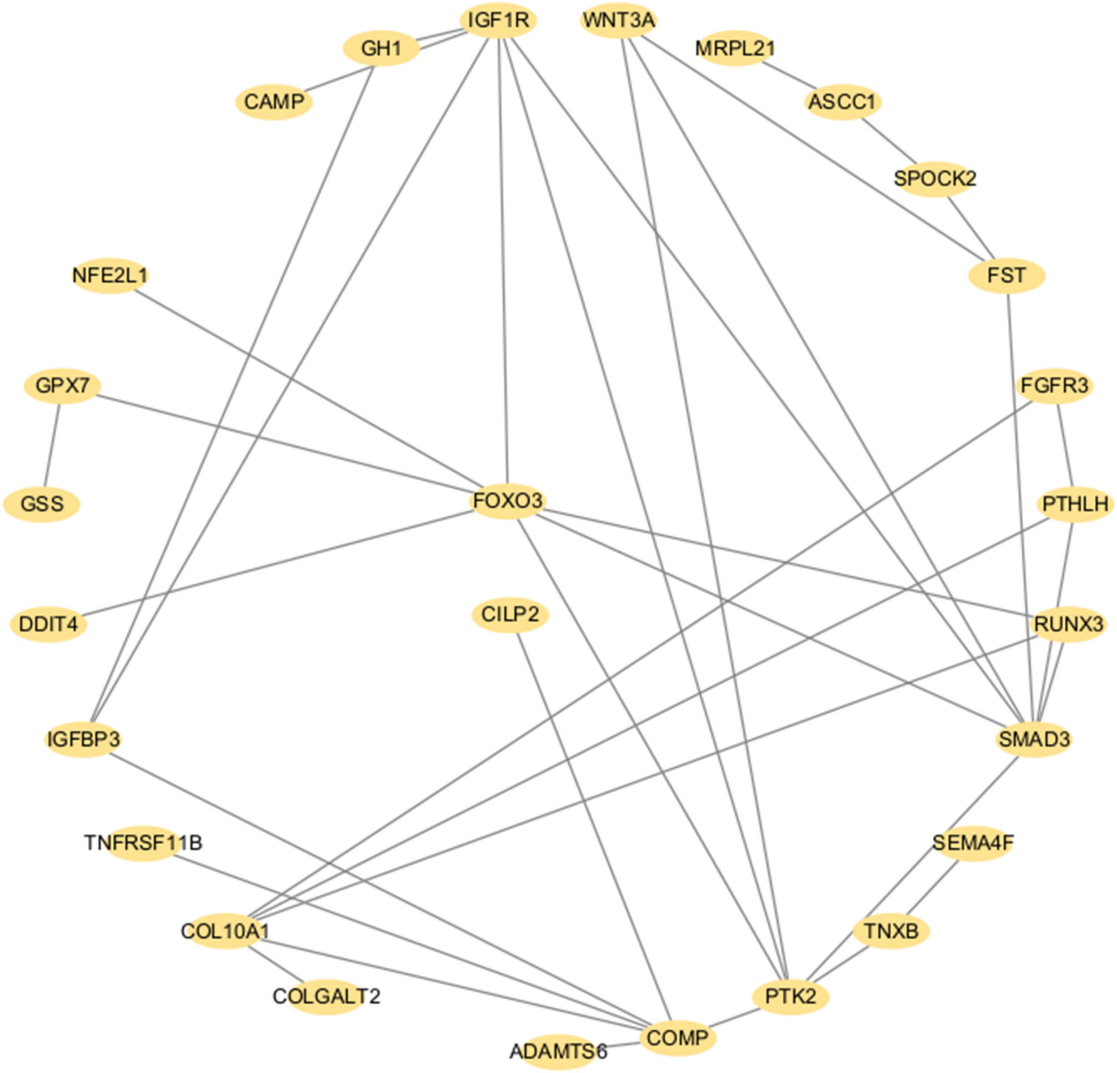
PPI network of the significant proteins associated with IDD identified via TWAS and PWAS analyses. Each dot represents a protein. Lines denote physical PPIs.

To elucidate the interactions among the candidate genes associated with IDD (TMEM190, CILP2, and FOXO3), we performed PPI analysis involving 88 proteins associated with IDD identified in TWAS and PWAS (Supplementary Data 1). There were 27 genes whose connections had interaction scores greater than 0.4. Notably, TMEM190 did not interact with either CILP2 or FOXO3. Although no direct interactions were observed between CILP2 and FOXO3, several core proteins—SMAD3, COMP, IGFBP3, IGF1R, COL10A1, RUNX3, and PTK2—were identified as mediators of the interaction between CILP2 and FOXO3 (Figure 6).

### Druggability of the identified genes and proteins

Among the genes identified through TWAS and eQTL analyses, 33 protein-coding genes were classified within the druggable genome: including 16 in tier 1, 8 in tier 2, and 9 in tier 3 (Supplementary Data 16). By searching the Open Target Platform, we identified several approved or investigational drugs targeting risk genes indicated by TWAS and eQTL analyses, including FGFR3, TGFA, CD79B, PDE3A, NQO1, AGER, ITGA2, CDK4, COL27A1, and PTK2 (Supplementary Data 17). Additionally, of the proteins identified through PWAS and pQTL analyses via ARIC or deCODE, 14 protein-coding genes were classified within the druggable genome: including 4 in tier 1 and 10 in tier 3 (Supplementary Data 18). We further identified several approved or investigational drugs targeting risk genes of IDD indicated by PWAS and pQTL analyses, namely PLG and PTHLH (Supplementary Data 19). Among the three potential causal genes, only CILP2 was druggable, while TMEM190 and FOXO3 were not in the druggable genome.

### Validation studies for potential causal genes of IDD with clinical samples and animal model

**FIGURE 7:**
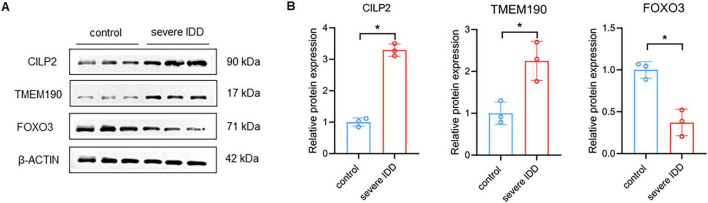
Validation of risk genes in clinical IVD tissues. **(A,B)** Western-blot analysis of TMEM190, CILP2, and FOXO3 in control and severe IDD patients. *n* = 3. Data represent the mean (SD). Two tailed *t*-test. *p < 0.05.

To explore the roles of *TMEM190*, *CILP2*, and *FOXO3* in IDD, we assessed their expression in human IVD specimens from mild degeneration (Grades I and II) and severe degeneration (Grades III, IV, and V). Western blot results showed increased expression of *TMEM190* and *CILP2* with concurrent decreased expression of *FOXO3* in severely degenerated IVD tissues (Figures 7A, B), aligning with findings in DEGs analysis.

**FIGURE 8:**
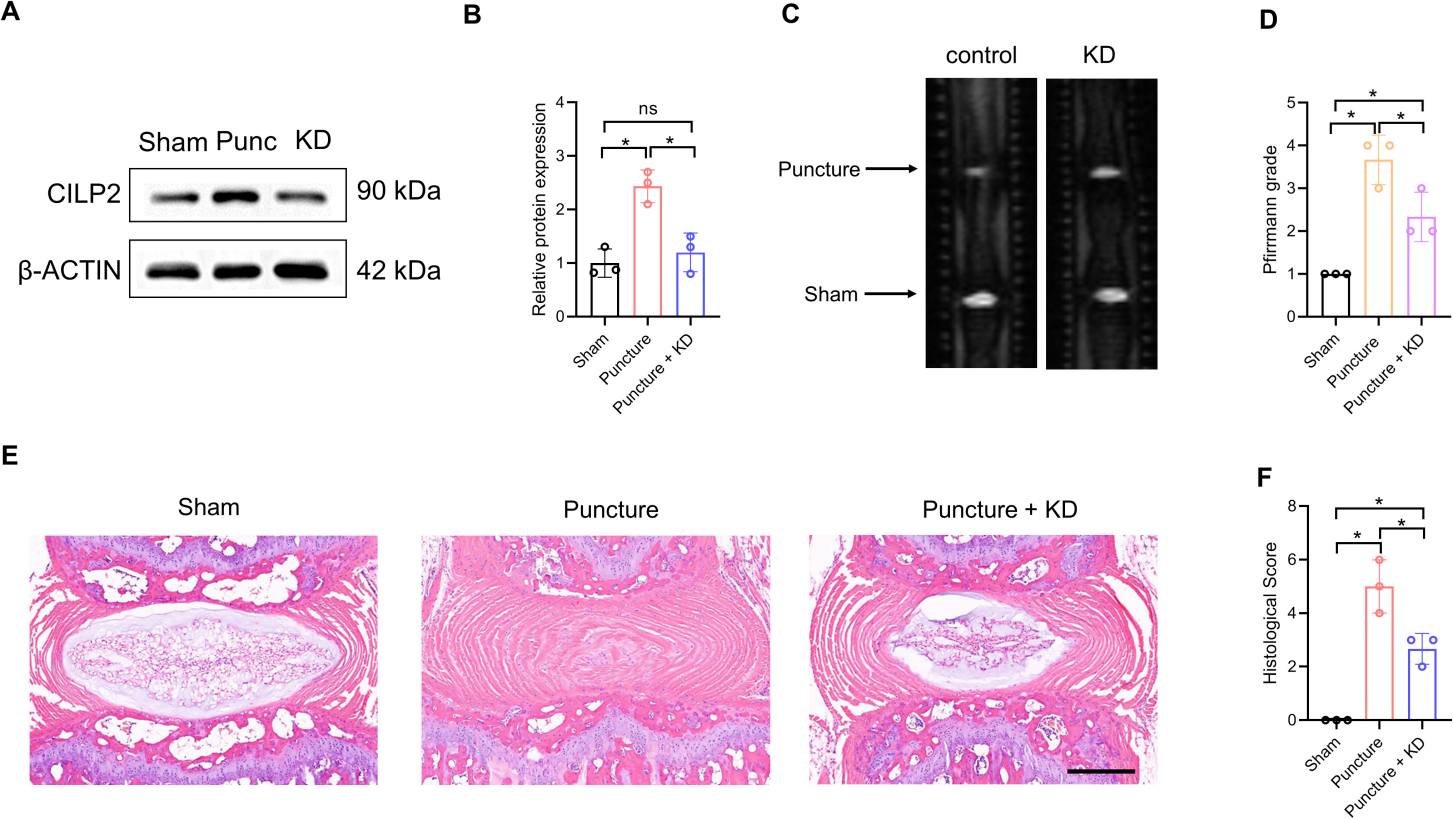
Down-regulation of CILP2 alleviated IDD progression in mouse model of IDD. **(A,B)** Western-blot analysis of CILP2 in Sham, puncture (Punc) and knocking down (KD) IVD tissues in mouse model of IDD. *n* = 3. **(C,D)** Magnetic resonance imaging (MRI) and Pfirrmann grades of IVD in mice treated as in **(A)**. *n* = 3. **(E,F)** Hematoxylin and Eosin (H&E) staining and histological score assessment of IVD in mice treated as in **(A)**. *n* = 3. Scale bar = 200 μm. Data represent the mean (SD). One-way ANOVA. *p < 0.05.

Given the combined evidence supporting CILP2, and its classification as a druggable target, we further investigated its role in IDD using a needle-induced IVD degeneration mouse model. As shown in Figures 8A, B, the protein level of CILP2 in the IVD was up-regulated after puncture treatment. However, this level decreased significantly following shRNA transfection. MRI examinations revealed that the grade score of the IVD was significantly increased after puncture treatment, while it decreased markedly with CILP2 knockdown (KD) (Figures 8C,D). H & E staining showed the degenerated progression was alleviated following the down-regulation of CILP2 (Figures 8E, F). These results suggest that down-regulation of CILP2 reduces the susceptibility to intervertebral disc degeneration progression in the mouse model of IDD.

## Discussion

To the best of our knowledge, this study is the first to employ multidimensional multi-omics data, including high-throughput genomics, whole-body transcriptomics, plasma proteomics and intervertebral disc transcriptomics, to investigate potential risk genes for IDD. Our integrative approach presented 104 TWAS-identified genes and 10 PWAS-identified proteins with IDD based on converging evidence supported by eQTL/pQTL colocalization analyses. These genes/proteins were enriched for key regulators of disc pathology, such as glycosphingolipid/sphingolipid metabolism. Three potential causal genes, *TMEM190*, *CILP2*, and *FOXO3*, were consistently supported by TWAS, two independent PWAS and colocalization analyses. These three genes were dysregulated in degenerated human discs, with *CILP2* further classified as druggable. We also validated the role of these causal genes, *TMEM190*, *CILP2* and *FOXO3* with clinical samples, as well as the role of CILP2 with animal model in IDD.

Glycosphingolipid/sphingolipid metabolism consistently emerged as a key pathway across all enrichment analyses of the identified associations. Both the TWAS and the PWAS results from the ARIC and deCODE cohorts strongly highlight this pathway as a critical factor in the pathogenesis of IDD. Sphingolipids, including ceramide and sphingosine-1-phosphate, constitute a major class of lipids found in all eukaryotic cells. Sphingolipids regulate a wide range of biological processes, including inflammation, mitochondrial function and apoptosis (32–34). The metabolic processes involved in sphingolipid biosynthesis and regulation were significantly enriched, underscoring their potential role in IDD. This pathway’s involvement in inflammation and apoptosis suggests that targeting sphingolipids synthesis could serve as a promising therapeutic strategy to alleviate disc disorders and associated pain.

Among the identified genes (*TMEM190*, *CILP2*, and *FOXO3*), *FOXO3* has been previously investigated in the context of IDD. FOXO3, a member of the forkhead box O transcription factor family, is known to regulate critical cellular processes, including the cell cycle, apoptosis, and metabolism, and is implicated in age-related diseases (35, 36). FOXO3 has been linked to IDD in numerous studies, where it functions as a mediator regulating the role of specific genes in the disease, such as YTHDF2 and P300 (37, 38). Furthermore, FOXO3 is involved in the molecular mechanisms of potential therapeutic agents for IDD, such as stem cell-derived exosomes and procyanidin C1, primarily by regulating oxidative stress (39, 40), which reinforces the potential of FOXO3 as therapeutic interventions for this condition. The current study found that FOXO3 was down-regulated in severely degenerated disc tissues, which aligns with previous findings (38, 41). Our study provides additional evidence for dysregulated FOXO3 expression in IDD. However, the contribution of FOXO3 to IDD still requires further exploration.

The roles of the other two identified genes, *TMEM190* and *CILP2*, in IDD are less well characterized. TMEM190 is located on chromosome 7 and contains five exons, which encode a small single-pass transmembrane protein (42). Small single-pass transmembrane proteins may be associated with mitochondrial oxidative phosphorylation (43), which has been linked to IDD. Additionally, TMEM190 may contribute to chondrocyte dedifferentiation (44). Given that cartilage endplate remodeling and altered chondrocyte subsets play key roles in IDD progression (45, 46), TMEM190 may involve in IDD pathogenesis. CILP2, a member of the cartilage intermediate layer protein family, encodes a matricellular protein predominantly expressed in cartilage cells but also in various other tissues (47). Quantitative proteomic analysis and immunohistochemistry have demonstrated increased *CILP2* levels in degenerated human intervertebral discs (48, 49). In current study, we found CILP2 was up-regulated in severe IDD tissues, which provides additional evidence that CILP2 play a role in IDD. Importantly, the inhibition of Cilp2 has been shown to improve mitochondrial dysfunction in sarcopenia via the WNT signaling pathway (47). Given the established roles of mitochondrial dysfunction and WNT signaling in IDD (50, 51), CILP2 is likely to play an important role in this condition. In the current study, we found that down-regulation of CILP2 alleviated IDD progression in mouse model of IDD. Our results provide more direct evidence for the role of CILP2 in the progression of IDD. Our multi-omics investigation and validation study with clinical samples and animal model offer evidence supporting the role of CILP2 as a disease-causing gene and therapeutic target in IDD. However, the functional mechanism of CILP2 in IDD was not explored in current study. It has been reported that CILP2 affect sarcopenia and hypertrophic scar by antagonizing Wnt signaling pathway (47), and reducing the ubiquitination of ACLY (52), respectively. Further research is warranted to elucidate the precise role of CILP2 in modulating IDD progression through these candidate signaling pathways.

While no direct interactions among the three genes (*TMEM190*, *CILP2* and *FOXO3*) were reported, GSEA revealed their collective involvement in the olfactory signaling pathway, and sensory perception. Notably, olfactory stem cells have been shown to exhibit a chondrogenic phenotype, promoting IVD regeneration in a rat model of disc injury (53), which indicates these three genes collectively contributed to the pathology of IDD. In addition, pain is a significant symptom of IDD. It has been reported that anti-sensory nerve transmission significantly suppresses inflammatory pain markers (54). The involvement of these three genes in sensory perception indicates that they share a similar pathway for the contribution of pain to IDD. To further explore the correlations, we performed a PPI analysis using data from TWAS and PWAS. Although no direct interactions were found among the three genes, several mediators of interaction between CILP2 and FOXO3 were identified, including COMP and IGFBP3, which have been linked to IDD progression (55, 56). These findings suggest that CILP2 and FOXO3 may collaboratively influence IDD through these mediators.

Our study has several strengths. Primarily, it integrates both genomic and proteomic data to provide comprehensive insights into the complex biological systems underlying IDD. Additionally, the validation of potential causal genes through two independent PWAS analyses strengthens the reliability of our findings. Furthermore, the datasets utilized, comprising extensive human transcriptomes, proteomes, and IDD GWAS data, are among the largest and most complete to date, enhancing the robustness of the results. Finally, we validated the risk genes derived from public datasets using clinical samples and animal model, which enhances the reliability of our findings.

Some limitations must be acknowledged. First, the PWAS for IDD utilized human blood proteome data; however, plasma proteins serve as systemic biomarkers and may not fully capture disc-specific changes, potentially introducing bias. Future studies should examine the proteomes specific to human intervertebral discs. Secondly, the identification of susceptible genes from a European database, coupled with clinical validation using specimens from the Chinese population, introduces population heterogeneity that may limit the generalizability of the findings. Future cross-ethnic validation studies should be conducted to assess the robustness of these findings across diverse populations and ensure their applicability in broader clinical contexts. Additionally, the mechanisms by which identified risk genes and the relevance of their enriched pathways contribute to IDD remain unclear, and additional studies are needed to further evaluate their potential as therapeutic targets. Besides, only the mouse tail disc puncture model was employed, which is an acute injury model and may not adequately replicate the chronic, progressive nature of human IDD. Also, the current transcriptomic samples predominantly represent European populations, whereas the proteomic samples are from American populations, and expanding the diversity of these datasets may yield more accurate estimations and broader applicability. Finally, gene-environment interactions and assortative mating could influence genetic effects and contribute to variance in the analysis. Unfortunately, due to the limitations inherent in the current dataset and the scope of the study design, it is not feasible to adjust for these factors in this particular analysis. Nevertheless, the strength of our study lies in its innovative integration of multi-omics data, which positions it as one of the first efforts to identify and validate novel genetic risk factors for IDD in such a comprehensive manner.

## Conclusion

In summary, we present an expanded resource of putatively causal genes associated with IDD, and highlight three novel potential causal genes (*TMEM190*, *CILP2*, and *FOXO3*). These findings provided a broad hint for further research on the potential mechanisms underlying IDD pathogenesis and highlight novel therapeutic targets for future investigations.

## Data Availability

All the data used in this study are publicly available without the need for special access. The specific sources of the data are as follows: TWAS Process: https://github.com/xqwen/fastenloc. IDD GWAS: https://pmc.ncbi.nlm.nih.gov/articles/PMC8810832. TWAS Prediction Model: https://predictdb.org/post/2021/07/21/gtex-v8-models-on-eqtl-and-sqtl/. fastENLOC Colocalization: Pre-computed GTEx multi-tissue eQTL annotations with hg38 position ID from https://github.com/xqwen/fastenloc. BLISS Software: Data calculations were performed using BLISS, which can be accessed at https://github.com/gcb-hub/BLISS. GEO database (GSE56081).

## Footnotes

1
https://www.ncbi.nlm.nih.gov/geo/query/acc.cgi?acc

2
https://platform.opentargets.org/

## Supplementary material

Supplementary Figure 1 GO enrichment analysis of the potential associated genes of IDD identified with TWAS. Each line represents a pathway with significance defined by an FDR-adjusted *p* < 0.05. The color intensity represents statistical significance. The dot size corresponds to the gene ratio, which is defined as the number of genes of a pathway to the total number of genes analyzed.

Supplementary Figure 2 GO enrichment analysis of the potential associated genes of IDD identified with PWAS via the ARIC dataset. Each line represents a pathway with significance defined by an FDR-adjusted *p* < 0.05. The color intensity represents statistical significance. The dot size corresponds to the gene ratio, which is defined as the number of genes of a pathway to the total number of genes analyzed.

Supplementary Figure 3 GO enrichment analysis of the potential associated genes of IDD identified with PWAS via the deCODE dataset. Each line represents a pathway with significance defined by an FDR-adjusted *p* < 0.05. The color intensity represents statistical significance. The dot size corresponds to the gene ratio, which is defined as the number of genes of a pathway to the total number of genes analyzed.
