# Supplementary material for: Identification and validation of novel risk genes for intervertebral disc disorder by integrating large-scale multi-omics analyses and experimental studies
Source: Front Med (Lausanne). 2025 Nov 12;12:1698050. doi: 10.3389/fmed.2025.1698050 (PMC12612748; doi:10.3389/fmed.2025.1698050)
Supplement: Supplementary file 2 [file Table_2.docx]

Supplementary Table 1 Basic clinical information of individuals with IDD

|  | I-II | III-V |
| --- | --- | --- |
| Number | 3 | 3 |
| Gender | 2 male and 1 female | 1 male and 2 females |
| Age （years） | 16.3±2.2 | 53.4±3.1 |
