# Supplementary material for: Identification and validation of novel risk genes for intervertebral disc disorder by integrating large-scale multi-omics analyses and experimental studies
Source: Front Med (Lausanne). 2025 Nov 12;12:1698050. doi: 10.3389/fmed.2025.1698050 (PMC12612748; doi:10.3389/fmed.2025.1698050)
Supplement: Supplementary file 3 [file Table_3.docx]

Supplementary Table 2 Number of colocalizing genes in TWAS positive and negative genes

|  | Colocalizing genes | Non-colocalizing genes | *p*-value |
| --- | --- | --- | --- |
| TWAS positive | 104 | 452 | *p* < 0.001 |
| TWAS negative | 42 | 16744 |  |

Chi-square test was used.
