# Supplementary material for: Identification and validation of novel risk genes for intervertebral disc disorder by integrating large-scale multi-omics analyses and experimental studies
Source: Front Med (Lausanne). 2025 Nov 12;12:1698050. doi: 10.3389/fmed.2025.1698050 (PMC12612748; doi:10.3389/fmed.2025.1698050)
Supplement: Supplementary file 4 [file Table_4.docx]

Supplementary Table 1 Basic clinical information of individuals with IDD for TMEM190, CILP2 and FOXO3 testing.

| Pfirrmann’s classification | I-II | III-V | *p* value |
| --- | --- | --- | --- |
| Number | 3 | 3 | - |
| Gender | 2 male and 1 female | 1 male and 2 females | - |
| Age (year) | 16.3±2.2 | 53.4±3.1 | *p* < 0.05 |
| BMI | 23.2±2.5 | 24.1±2.3 | *p* >= 0.05 |
| Symptom duration (month) | 2.3±0.7 | 6.3±2.1 | *p* < 0.05 |
