# Supplementary figures and images for: Identification and validation of novel risk genes for intervertebral disc disorder by integrating large-scale multi-omics analyses and experimental studies
Source: Front Med (Lausanne). 2025 Nov 12;12:1698050. doi: 10.3389/fmed.2025.1698050 (PMC12612748; doi:10.3389/fmed.2025.1698050)

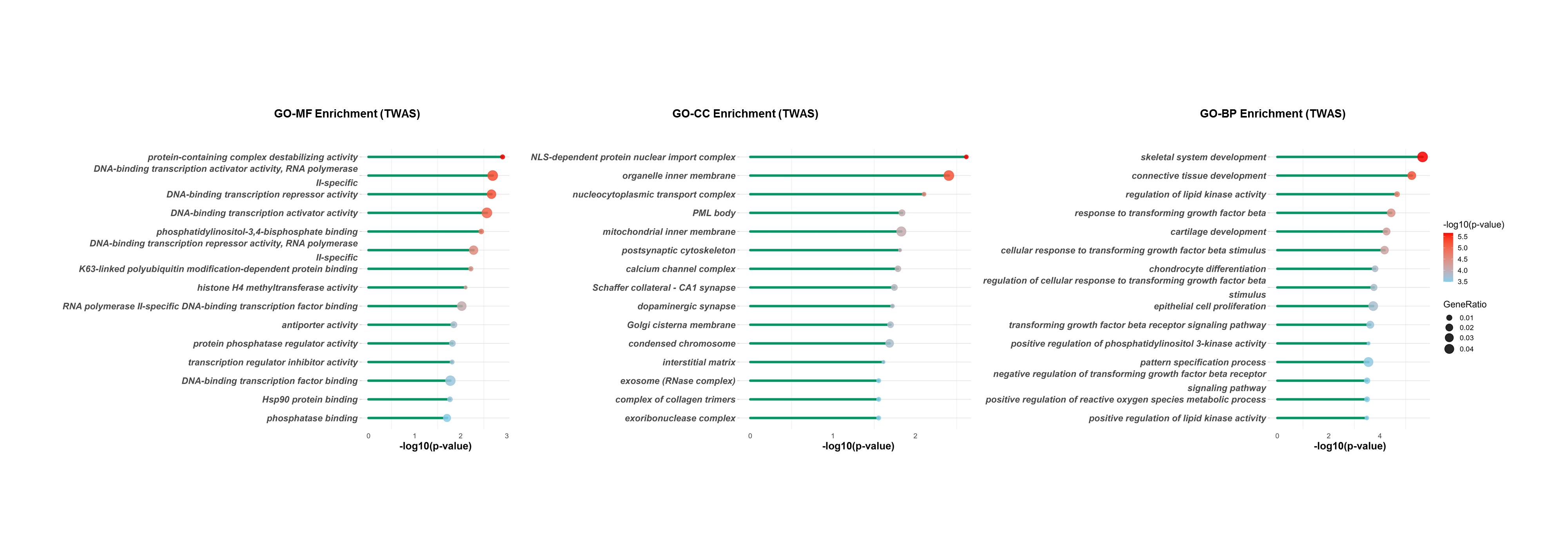

Supplement: Supplementary Figure 1 — GO enrichment analysis of the potential associated genes of IDD identified with TWAS. Each line represents a pathway with significance defined by an FDR-adjusted p < 0.05. The color intensity represents statistical significance. The dot size corresponds to the gene ratio, which is defined as the number of genes of a pathway to the total number of genes analyzed. [file Image_1.tif]

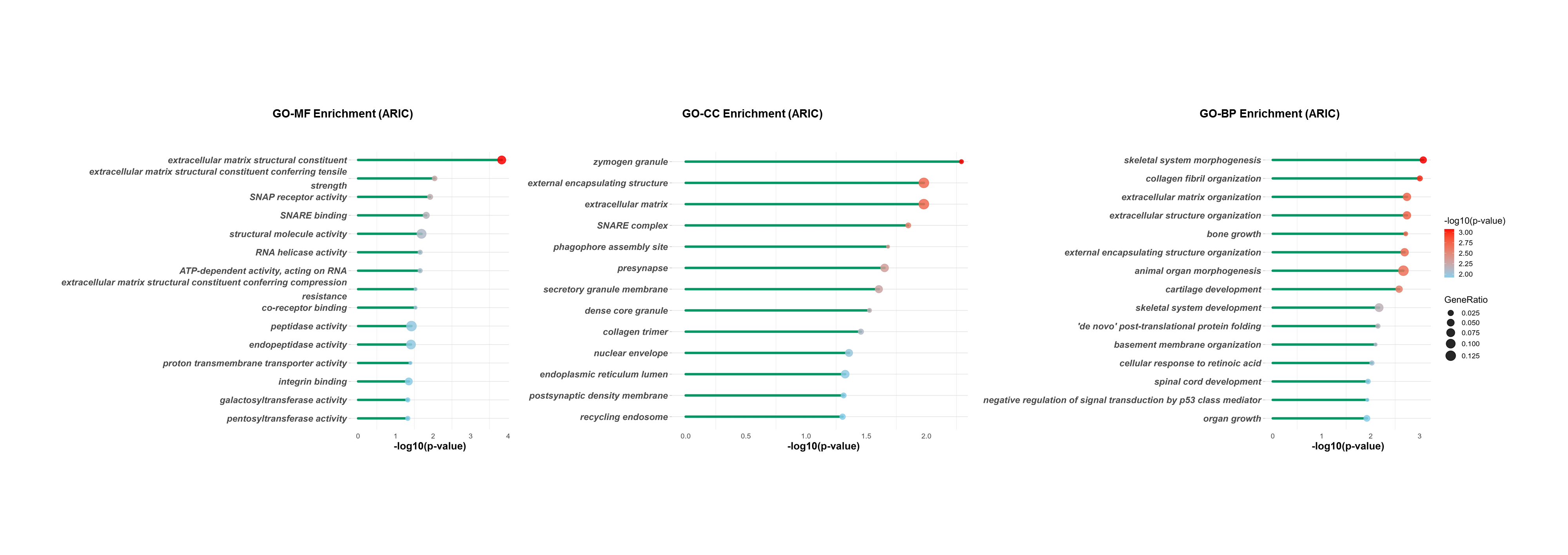

Supplement: Supplementary Figure 2 — GO enrichment analysis of the potential associated genes of IDD identified with PWAS via the ARIC dataset. Each line represents a pathway with significance defined by an FDR-adjusted p < 0.05. The color intensity represents statistical significance. The dot size corresponds to the gene ratio, which is defined as the number of genes of a pathway to the total number of genes analyzed. [file Image_2.tif]

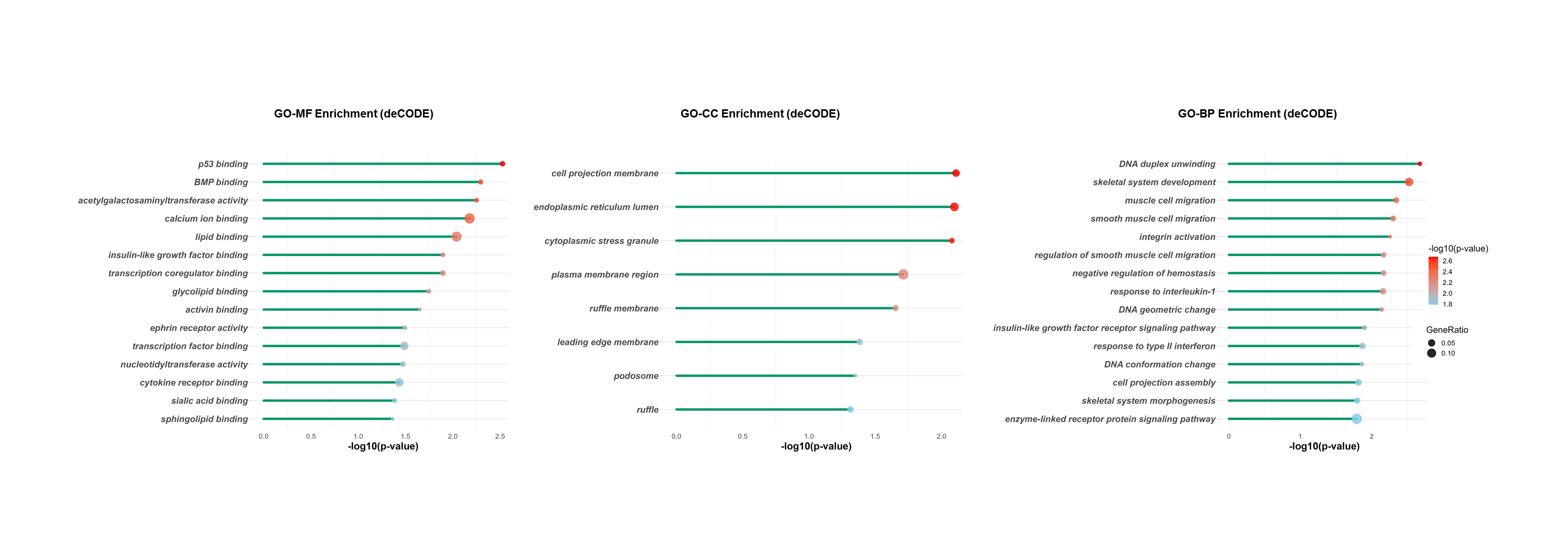

Supplement: Supplementary Figure 3 — GO enrichment analysis of the potential associated genes of IDD identified with PWAS via the deCODE dataset. Each line represents a pathway with significance defined by an FDR-adjusted p < 0.05. The color intensity represents statistical significance. The dot size corresponds to the gene ratio, which is defined as the number of genes of a pathway to the total number of genes analyzed. [file Image_3.tif]
